# Single-Pulse TMS to the Temporo-Occipital and Dorsolateral Prefrontal Cortex Evokes Lateralized Long Latency EEG Responses at the Stimulation Site

**DOI:** 10.3389/fnins.2021.616667

**Published:** 2021-03-12

**Authors:** Tomasz A. Jarczok, Friederike Roebruck, Lena Pokorny, Lea Biermann, Veit Roessner, Christoph Klein, Stephan Bender

**Affiliations:** ^1^Department of Child and Adolescent Psychiatry, Psychosomatics, and Psychotherapy, Faculty of Medicine and University Hospital Cologne, University of Cologne, Cologne, Germany; ^2^Department of Child and Adolescent Psychiatry, Faculty of Medicine, TU Dresden, Dresden, Germany; ^3^Clinic for Child and Adolescent Psychiatry, Medical Faculty, University of Freiburg, Freiburg, Germany; ^4^Department of Psychiatry, Medical School, National and Kapodistrian University of Athens, Athens, Greece

**Keywords:** transcranial magnetic stimulation (TMS), electroencephalography (EEG), TMS-EEG, dorsolateral prefrontal cortex, temporo-occipital cortex, N100, lateralized readiness potential (LRP)

## Abstract

**Introduction:**

Transcranial magnetic stimulation (TMS)–evoked potentials (TEPs) allow for probing cortical functions in health and pathology. However, there is uncertainty whether long-latency TMS-evoked potentials reflect functioning of the targeted cortical area. It has been suggested that components such as the TMS-evoked N100 are stereotypical and related to nonspecific sensory processes rather than transcranial effects of the changing magnetic field. In contrast, TEPs that vary according to the targeted brain region and are systematically lateralized toward the stimulated hemisphere can be considered to reflect activity in the stimulated brain region resulting from transcranial electromagnetic induction.

**Methods:**

TMS with concurrent 64-channel electroencephalography (EEG) was sequentially performed in homologous areas of both hemispheres. One sample of healthy adults received TMS to the dorsolateral prefrontal cortex; another sample received TMS to the temporo-occipital cortex. We analyzed late negative TEP deflections corresponding to the N100 component in motor cortex stimulation.

**Results:**

TEP topography varied according to the stimulation target site. Long-latency negative TEP deflections were systematically lateralized (higher in ipsilateral compared to contralateral electrodes) in electrodes over the stimulated brain region. A calculation that removes evoked components that are not systematically lateralized relative to the stimulated hemisphere revealed negative maxima located around the respective target sites.

**Conclusion:**

TEPs contain long-latency negative components that are lateralized toward the stimulated hemisphere and have their topographic maxima at the respective stimulation sites. They can be differentiated from co-occurring components that are invariable across different stimulation sites (probably reflecting coactivation of peripheral sensory afferences) according to their spatiotemporal patterns. Lateralized long-latency TEP components located at the stimulation site likely reflect activity evoked in the targeted cortex region by direct transcranial effects and are therefore suitable for assessing cortical functions.

## Introduction

Since the introduction of transcranial magnetic stimulation (TMS) (Barker et al., 1985), there have been considerable efforts to extend its scope as a clinical and research tool. Repetitive TMS (rTMS) is used in the clinical treatment of depression (Perera et al., 2016). Also, rTMS (George, 2019) and other brain stimulation techniques such as trancranial direct current stimulation (tDCS) (Venkatasubramanian and Narayanaswamy, 2019) are increasingly evaluated as experimental treatments in a variety of neuropsychiatric conditions. The combination of TMS with concurrent electroencephalography (TMS-EEG) allows for the measurement of neural activity resulting directly from the TMS procedure with high temporal resolution in both motor and non-motor cortical regions (Cracco et al., 1989; Ilmoniemi et al., 1997; review in: Tremblay et al., 2019). In the context of neuropsychiatric disorders, TMS-EEG has been used to measure cortical excitability in functionally relevant brain areas such as the primary motor cortex (M1) (Bruckmann et al., 2012) and the dorsolateral prefrontal cortex (DLPFC) (Noda et al., 2017; Voineskos et al., 2018) in attempts to identify biomarkers for cortical dysfunctions. Therapeutic neuromodulation of cortical excitability through brain stimulation techniques could potentially be made more effective if it was possible to measure the activity and monitor the functional changes in the targeted brain region throughout the treatment course. For example, rTMS to the DLPFC for the treatment of depression may benefit from the possibility to measure and monitor the excitability of the target cortical area with TMS-EEG. However, despite promising attempts to monitor the effects of rTMS and tDCS using TMS-EEG (Helfrich et al., 2012; Moliadze et al., 2018; Alyagon et al., 2020), there is no clear consensus among researchers about which TMS-EEG parameters reflect functions of the targeted brain region. This hinders the further development of TMS-EEG basic research and its translation into clinical practice.

In TMS-EEG, the EEG signal time-locked to the TMS pulse is averaged to obtain TMS-evoked potentials (TEPs). TEP deflections reflect the activity of the targeted populations of neurons resulting from transcranial effects of the changing magnetic field and secondary activation of other functionally connected neurons (transcranially evoked activity). However, TMS also indirectly evokes cortical activity through the unintended activation of sensory peripheral nerves (sensory evoked activity) including auditory activity associated with the coil click and somatosensory activity caused by activation of afferent cranial nerves (Gordon et al., 2018; Conde et al., 2019). Yet, while compound TEPs are a summation of several neural processes, there is no consensus regarding the spatiotemporal pattern reflecting the actual transcranially evoked activity.

The second prominent negative TEP peak, often referred to as TMS-evoked N100 in motor cortex and DLPFC stimulation, is one of the most robust and often studied TEP peaks (Nikulin et al., 2003; Bender et al., 2005; Bonato et al., 2006; Premoli et al., 2014; Rogasch et al., 2015; Du et al., 2017). It is the TEP deflection with the highest retest reliability (Kerwin et al., 2017). The N100 in TMS applied to M1 has a lateralized maximum over the ipsilateral M1 (Paus et al., 2001; Bender et al., 2005; Bonato et al., 2006), is modulated by the activational state of M1 (Bruckmann et al., 2012) and can be used to successfully monitor excitability changes resulting from rTMS of M1 (Helfrich et al., 2012). These findings are consistent with the notion that the N100 is site-specific and reflects local intracortical excitability–inhibition networks in the targeted brain region. By contrast, other studies found the TMS-evoked N100 to be uniform across several different stimulated brain areas with a stereotypical symmetrical distribution over the vertex irrespective of the targeted cortex region, therefore interpreting it as an unspecific response representing global properties of the brain or even an artifact (Du et al., 2017; Freedberg et al., 2020). In order to use TEPs in neuropsychiatric research and to adequately translate findings into applications as a neurostimulation biomarker, it is crucial to determine which TEP components reflect local cortical properties evoked by direct transcranial effects. Evoked components with a lateralized site-specific topography (i.e., varying with the stimulated brain region) are most likely transcranially evoked (Conde et al., 2019) and would thus be suitable parameters to study cortical excitability.

Therefore, we studied the spatiotemporal distribution of TEPs during the stimulation of the temporo-occipital cortex (TOC) and the DLPFC of both hemispheres. Although there is still uncertainty regarding late deflections (>80 ms), early TEPs (<80 ms) are more widely recognized to reflect activity of the stimulated cortex (Herring et al., 2015; Du et al., 2017; Conde et al., 2019; Rogasch et al., 2020). We thus focused on late negative deflections corresponding to the N100 in motor cortex stimulation and expected to identify lateralized site-specific components over the stimulated brain region.

## Materials and Methods

### Ethics Statement

The study protocols were approved by the Ethics Committee of the Faculty of Medicine, University of Cologne, Germany, for DLPFC stimulation (document no. 15-432) and the Ethics Committee of the Technical University Dresden, Germany, for TOC stimulation (document no. EK 184052011). All participants provided written consent after being informed about the study.

### Experimental Design

We integrated the samples of two separate studies. One sample received TMS to the TOC; the other sample received TMS to the DLPFC. For both targeted brain areas, TMS was performed over the left and the right hemisphere sequentially in a counterbalanced order. A quantitative assessment of hemispheric lateralization of TEPs in the stimulated brain region was accomplished through within-subject comparison of left- versus right-sided TMS. As there were some methodological differences between the two studies, we did not intend to make any direct quantitative comparisons (e.g., amplitude differences) between TOC and DLPFC TMS. Therefore, only major differences in the topographies of lateralized TEP (LatTEP) components that cannot be explained by differences between the subjects or methods of the two studies are reported.

### Subjects

Participants were healthy adults who reported no history of neurological or psychiatric disorders and were free of medication at the time of testing. Before participation we screened for exclusion criteria according to established safety guidelines (Rossi et al., 2009). Persons with epilepsy in close relatives were also excluded for safety reasons. The TOC stimulation sample included 17 subjects (mean age, 24.7 ± 6.1 years; 11 female, 6 male subjects; mean, IQ 113.4 ± 9.1). The DLPFC stimulation sample included 26 subjects (mean age, 22.6 ± 1.8 years; 23 female and 3 male; mean IQ, 115.1 ± 10.1). All participants were right-handed according the Edinburgh Handedness Inventory (Oldfield, 1971).

### Electroencephalography

A 64-channel DC-EEG was recorded concurrently with a TMS procedure. The EEG signal was amplified by a BrainAmp DC amplifier and recorded with a sampling rate of 5,000 Hz using the BrainVision Recorder 1.20 (both Brain Products, München, Germany). Custom-made EEG caps, which were equipped with TMS-compatible Ag/Ag-Cl electrodes, were used for both TOC and DLPFC (Easycap GmbH, Herrsching, Germany). Electrodes were arranged in equidistant montages on five concentric rings around Cz with electrodes on the horizontal and vertical central line corresponding to the 10–10 system (Chatrian et al., 1985). Other electrodes were named according to the nearest corresponding electrodes in the 10–10 system. Electrode layouts of caps used for TOC and DLPFC were identical, except for additional bilateral supraorbital electrodes and an electrode at the nasion for DLPFC stimulation. For TOC stimulation, Fpz served as reference electrode, whereas for DLPFC stimulation, Cz served as reference electrode during recording. EEG data were re-referenced to an average reference offline, in order to ensure independence of topographies from the reference electrode. Impedances were kept below 10 kΩ.

### Transcranial Magnetic Stimulation

For TOC stimulation, the TMS procedure was performed using a PowerMAG 100 Stimulator (Mag & More GmbH, München, Germany) with a figure-of-8 coil with an outer diameter of each wing of 70 cm. As the procedure was performed as part of an experiment in which TMS was used to perturb visual working memory processes, the exact placement of the coil was individually determined resulting in some interindividual variation of the locus of stimulation. The site was determined by localizing the visual N700 event-related potential component reflecting visual working memory processes (Bender et al., 2008). The targeted region was thus in secondary visual areas (V2) located in lower parts of the occipital lobe bordering the temporal lobe (visual “what” pathway) (Clark et al., 2010). The exact procedure used to determine the locus of stimulation is described in the Supplementary Material. In all subjects, the locus of stimulation was located between P7 and P11 for left-sided TMS and between P8 and P12 for right-sided TMS. The interindividual variation of the stimulation location had only a small nonsystematic effect on the TEP topography (Supplementary Figures 1, 2). The TEPs recorded at the homologous electrodes P9 and P10 were used for further analysis, which best reflected the grand average topographic maximum for the two stimulation sides.

During the stimulation procedure, the coil was held manually by a trained examiner. The coil was placed tangentially to the skull over the stimulated region. The stimulator was externally triggered by a PC running Presentation software 18.1 (NeuroBehavioral Systems, Berkley, CA, United States), which generated transistor–transistor–logic triggers that were also registered in the recording software. A total of 20 TMS single pulses were administered over each hemisphere. High reliability of the data indicated a sufficient signal-to-noise ratio with the amount of trials (see section “*Preprocessing”* and Supplementary Table 1 and Supplementary Figure 1). The interstimulus intervals varied evenly between 5 and 7 s (mean, 6 s). The participants were instructed to sit upright and still in a chair and to fixate a cross located on a computer screen in front of them in order to reduce movement and eye artifacts.

For DLPFC stimulation, the TMS procedure was applied using a MagPro X100 MagOption stimulator and a figure-of-8 coil with a diameter of 2 × 75 mm (MagVenture, Farum, Denmark). The coil was placed over electrodes F5 for left-sided stimulation and F6 for right-sided stimulation as this method has been recommended as the most accurate to target the DLPFC when individual structural MRI data are not available (Rusjan et al., 2010). The coil was held manually by a trained examiner. Like for TOC stimulation, the stimulator was triggered by the Presentation software. The protocol encompassed a total of 45 TMS single pulses for each hemisphere with interstimulus intervals varying evenly between 5 and 8 s (mean, 6.5 s). High reliability of the data indicated a sufficient signal-to-noise ratio with the amount of trials (see section “*Preprocessing”* and Supplementary Table 1 and Supplementary Figure 1). The participants were instructed to sit upright and still in a chair and to fixate a cross located on a computer screen.

The stimulation intensity for the stimulation protocol in both groups was set to 120% of resting motor threshold (RMT). To measure the individual RMT in both groups, an electromyogram was recorded from the first dorsal interosseus muscle of the contralateral hand with self-adhesive electrodes (H207PG/F; Covidien, Mansfield, MA, United States). The active electrode was placed over the first dorsal interosseus muscle; the reference electrode was placed over the basic phalanx of digit III for DLPFC and the proximal interphalangeal joint of digit II for TOC. Motor-evoked potentials (MEPs) were amplified with a Brain Amp ExG MR amplifier (Brain Products, München, Germany). Single pulses were applied at the position over the left primary motor cortex where the most consistent and highest MEP peak-to-peak amplitudes were recorded (hot spot). For TMS to the TOC, RMT was defined as the intensity that evoked an MEP of over 50 μV in 5 of 10 stimuli at the hot spot. For the DLPFC, RMT was determined by applying single TMS pulses at the hot spot in varying intensities according to the maximum likelihood method (Awiszus, 2003) using the software TMS Motor Threshold Assessment Tool (MTAT 2.0^1^). Mean RMT was 65.9% ± 7.0% stimulator output for TOC and 51.6 ± 10.0 stimulator output for DLPFC. As TEP amplitudes are affected by the stimulation intensity, a comparison of amplitudes across groups is not possible, and only amplitude comparison within subjects can be interpreted. Notably, shifts of topographies do not result from changes of stimulation intensities.

Previous studies suggesting that TMS evokes invariable potentials located at the vertex were performed without white noise or somatosensory masking (Du et al., 2017). Also, it is uncertain whether masking procedures can eliminate sensory input completely from the overall evoked potentials (Biabani et al., 2019; Conde et al., 2019; Siebner et al., 2019). As our aim was to identify lateralized site-specific components in compound TEPs including sensory activity, we performed TMS without masking procedures.

### EEG Data Analysis

#### Preprocessing

The EEG was analyzed offline with Brain Vision Analyzer 2.1 software (Brain Products, München, Germany). The EEG data were re-referenced to the average reference. The sampling rate was reduced to 500 Hz. As down-sampling in Brain Vision Analyzer includes an automatic filtering process (low-pass filter 225 Hz, 24 db/oct), a slight broadening of the high amplitude TMS pulse artifact occurred. In order to prevent a contamination by the pulse artifact, the time segments from −10 to 40 ms in TOC stimulation and from −10 to 20 ms in DLPFC stimulation around the TMS pulse were removed and then linearly interpolated (Thut et al., 2011; different time segments were interpolated, because the duration of the high-amplitude TMS artifact differed slightly between groups). The EEG was then segmented into epochs of −500 to 500 ms relative to the TMS pulse. A baseline correction procedure was performed with the interval of −110 to −10 ms serving as the baseline (the last 10 ms before the onset of TMS were not included in the baseline to exclude contamination of the baseline by a distortion of the TMS artifact). Epochs were visually inspected for artifacts and were removed if artifacts severely affected further analysis of the segment. Further artifacts were subsequently removed in an independent component analysis. Later, linear DC trends were removed. All available epochs were averaged to create TEPs.

As the amount of trials per condition was different across the two stimulated brain regions, we assessed the reliability of TEPs to establish that the signal-to-noise ratio was sufficient. To this end, we calculated averages for odd and even TMS trials separately. Preprocessing and peak measurements were performed using the same methodology as reported for the overall TEP averages. The intraclass correlation coefficients for odd and even trials were found to be very high (Supplementary Table 1) (Cicchetti, 1994). The time courses and topographies of odd and even trials were highly consistent in all stimulation conditions (Supplementary Figure 3), indicating a sufficient signal-to-noise ratio.

#### LatTEP Analysis

In order to test our hypothesis that TMS evokes activity localized at the stimulation site, we aimed at extracting systematically lateralized activity from the TEPs. To this end, we performed a calculation analogous to the lateralized readiness potential (LRP) (Coles, 1989; Eimer, 1998) with TEPs of homologous electrodes for both stimulation sides. The signals of each pair of homologous electrodes for both stimulation sides are used to calculate a single measure named LatTEP, e.g., for homologous electrode pairs F5 and F6: LatTEP F5/F6 = [F5(TMS left) - F6(TMS left) + F6(TMS right) - F5(TMS right)]/2 (analogous to Coles, 1989). The channels resulting from the LatTEP calculation were named LatTEP P9/P10 (temporo-occipital brain region) and LatTEP F5/F6 (frontal brain region). This procedure integrates measurements over both hemispheres (i.e., ipsilateral electrodes and homologous contralateral electrodes) for TMS to both sides. It eliminates processes that are either symmetrical to the midline or asymmetrical but localized in the same hemisphere irrespective of the side of stimulation (e.g., left-sided preponderance for both left- and right-sided TMS). The procedure retains systematically lateralized activity from the original evoked potentials, i.e., activity that changes hemispheres depending on the side of stimulation.

#### Peak Detection

We aimed at measuring the peak amplitude of the long-latency negative peak of the TEP. Peaks were detected automatically and confirmed by visual inspection in both regular TEPs and LatTEPs. In order to determine the search window for peaks, we inspected the grand average latencies at electrodes overlying the respective site of stimulation and compared the results with latencies reported in the literature.

For DLPFC stimulation, we searched for the maximum amplitude in the time window from 80 to 140 ms following the TMS pulse in agreement with previous reports (Lioumis et al., 2009; Kerwin et al., 2017). As LatTEP latencies tended to be shorter, a slightly broader peak detection window of 60–140 ms was used for LatTEPs. For TOC stimulation, the second prominent peak showed a markedly longer peak latency and a broader peak, which was in agreement with previous studies (Rosanova et al., 2009; Herring et al., 2015; Samaha et al., 2017; Belardinelli et al., 2019). We thus searched for the maximum amplitude in the time window from 140 to 230 ms. Peak latencies were determined in the reference channel overlying the site of stimulation for each stimulation condition (F5: left DLPFC, F6: right DLPFC, P9: left TOC, P10: right TOC). For the analysis of LatTEPs, the reference channels LatTEP F5/F6 for DLPFC and LatTEP P9/P10 for TOC were used. Amplitudes in all electrodes were measured at this peak latency ± 10 ms of the respective stimulation condition in all analyzed channels. For the comparison of amplitudes across stimulation sites, we used the amplitudes of all channels overlying the stimulation sites in one of the four stimulation conditions (F5, F6, P9, P10). Additionally, amplitudes in electrode Cz were analyzed as a control location, since a topographic maximum at the vertex has previously been reported (Du et al., 2017). As we analyzed a negative deflection, we henceforth use the term *higher amplitudes* to refer to higher negative voltage values.

### Statistical Analysis

Statistical analyses were performed using the IBM SPSS Statistics versions 23 and 25 (IBM Corp., Armonk, NY, United States).

TEPs were screened for outliers (>3 standard deviations from the mean), and the Shapiro–Wilk test was used to test for a normal distribution of the data. For DLPFC stimulation, TEP amplitudes included two outliers that caused a violation of normality. These were a result of artifacts that could not be removed adequately through the artifact rejection procedure. After the removal of the two subjects, all variables were normally distributed. The removal of the two subjects did not induce any systematic effects and did not, in particular, produce the presented results. In the TOC stimulation condition, there were no outlier values, and all parameters were normally distributed.

We tested whether TEP peaks and LatTEP peaks localized at the stimulation sites were significantly different from the baseline with a one-sample *t*-test against the value 0. For TOC and DLPFC stimulation, repeated-measures analyses of variance (ANOVAs) were calculated to test whether the maximum of the TEP was localized at the site of stimulation. The two separate ANOVAs with the dependent variables N100 and N180 amplitudes included the factors TMS SIDE (TMS applied to left side vs. TMS applied to right side), HEMISPHERE (left hemisphere electrodes vs. right hemisphere electrodes), and BRAIN REGION (temporo-occipital electrodes/P9 and P10 vs. frontal electrodes/F5 and F6).

To compare amplitudes at the respective site of stimulation to amplitudes at electrode Cz, repeated-measures ANOVAs were conducted for each dependent variable (N100 and N180 amplitudes) with the factors, TMS SIDE and ELECTRODE LOCALIZATION (factor levels: “electrode at the site of stimulation” and “electrode Cz”).

In order to compare LatTEP amplitudes in the stimulated vs. the non-stimulated cortical region, repeated-measures ANOVAs with the dependent variables LatTEP N100 and LatTEP N180 amplitudes and the factor BRAIN REGION (levels: LatTEP F5/F6 vs. LatTEP P9/P10) were conducted for TOC and DLPFC stimulation.

Significant interaction effects were followed up by further ANOVAs of reduced complexity.

## Results

### Temporo-Occipital Stimulation

#### TEP Time Course

**FIGURE 1:**
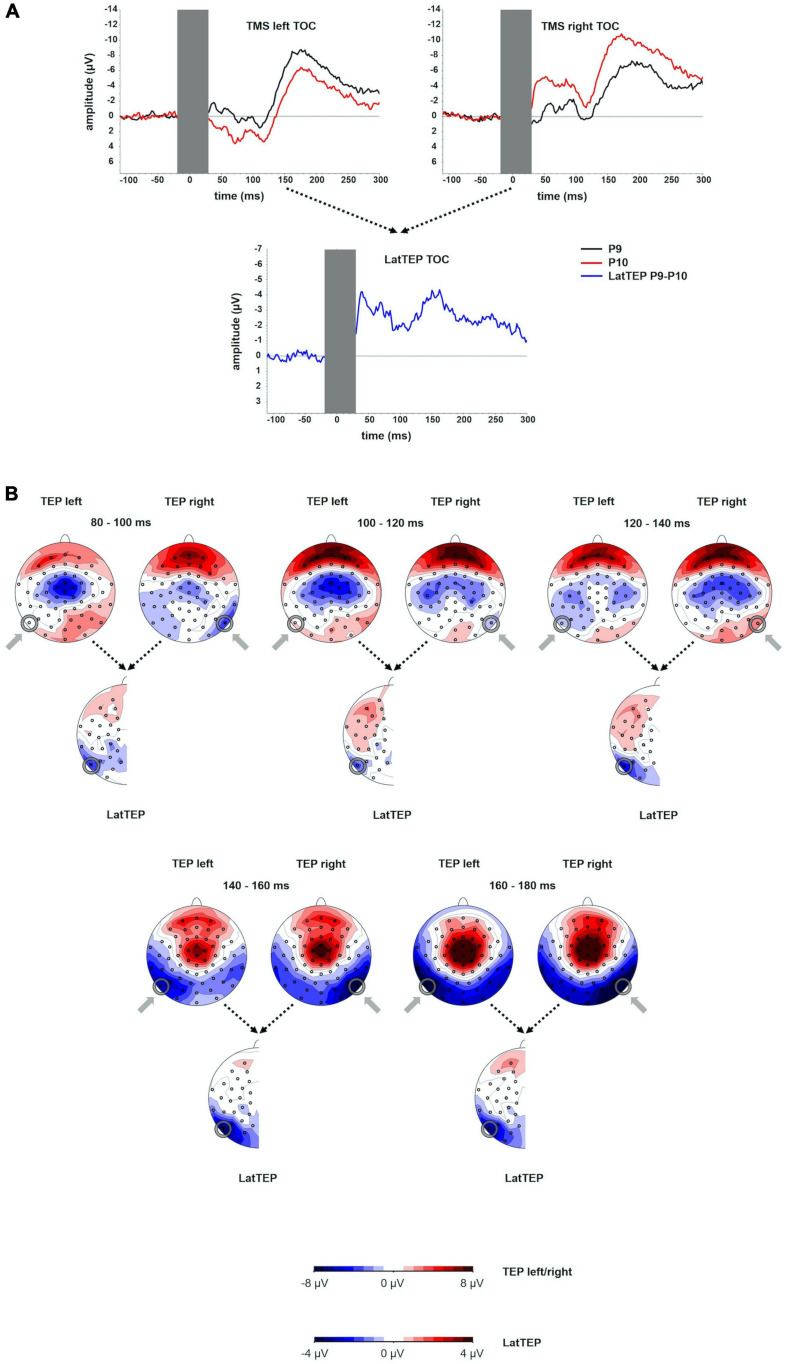
**(A)** TEP time course at electrodes P9 and P10 for TMS to the left (TMS left TOC) and the right (TMS right TOC) temporo-occipital cortex. The extent to which TEPs are higher (more negative) ipsilateral than contralateral to the side of stimulation is reflected in LatTEP amplitudes. Lateralization of evoked activity from both stimulation sides is condensed in one measure (LatTEP P9/P10). The LatTEP peaks at approximately 170 ms after the TMS pulse. Note the different scaling of the *y* axis between TEPs and LatTEPs. **(B)** Topographical plots of TEPs in time segments of 20-ms length for TMS to the left (TMS left TOC) and right (TMS right TOC) temporo-occipital cortex. LatTEP topographies are derived from TEP maps of both stimulation sides with each channel calculated according to the LatTEP formula. LatTEP maps show a topographical maximum around electrode LatTEP P9/P10 seen most prominently in the time range from 140 to 180 ms. Note that the color-coding scales differ between TEPs and LatTEPs.

The TEP time course in the electrodes overlying the sites of stimulation showed a first negative deflection at approximately 40 ms, a positive deflection peaking at approximately 110 ms and a more prominent and broader negative deflection peaking at approximately 180 ms (N180). The amplitude of the N180 at the site of stimulation was significantly different from the baseline [at electrode P9 for left TMS: *t*(16) = −5.72; *p* < 0.001; at electrode P10 for right TMS: *t*(16) = −9.37; *p* < 0.001]. The LatTEP time course showed a negative deflection with a peak at approximately 40 ms and another prominent negative peak at approximately 170 ms (LatTEP N180) (Figure 1A). LatTEP N180 amplitude at electrode LatTEP P9/P10 was significantly different from the baseline [*t*(16) = −5.60; *p* < 0.001].

#### TEP Topography

In the topographical distributions of the TEPs, there was a pronounced negativity around electrode P9 for left-sided TMS and around P10 for right-sided TMS, which is visible most clearly in the time window from 140 to 180 ms. In the time window from 80 to 120 ms, a central symmetrical negativity (located around Cz and FCz) was present. Furthermore, there was a symmetrical positivity located at the vertex and a broad posterior negativity visible in the time range from 140 to 180 ms. To the extent that this activity is identical in homologous electrodes of both hemispheres (i.e., symmetrical to the midline) for both stimulation sides, it is canceled out in LatTEPs. LatTEPs show a posterior negativity with a clear maximum around electrode LatTEP P9/P10 in the same time window. No prominent lateralized negativity was found over other brain regions (Figure 1B).

#### Lateralized Site-Specific Activity at the Stimulation Site for TOC TMS

**FIGURE 2:**
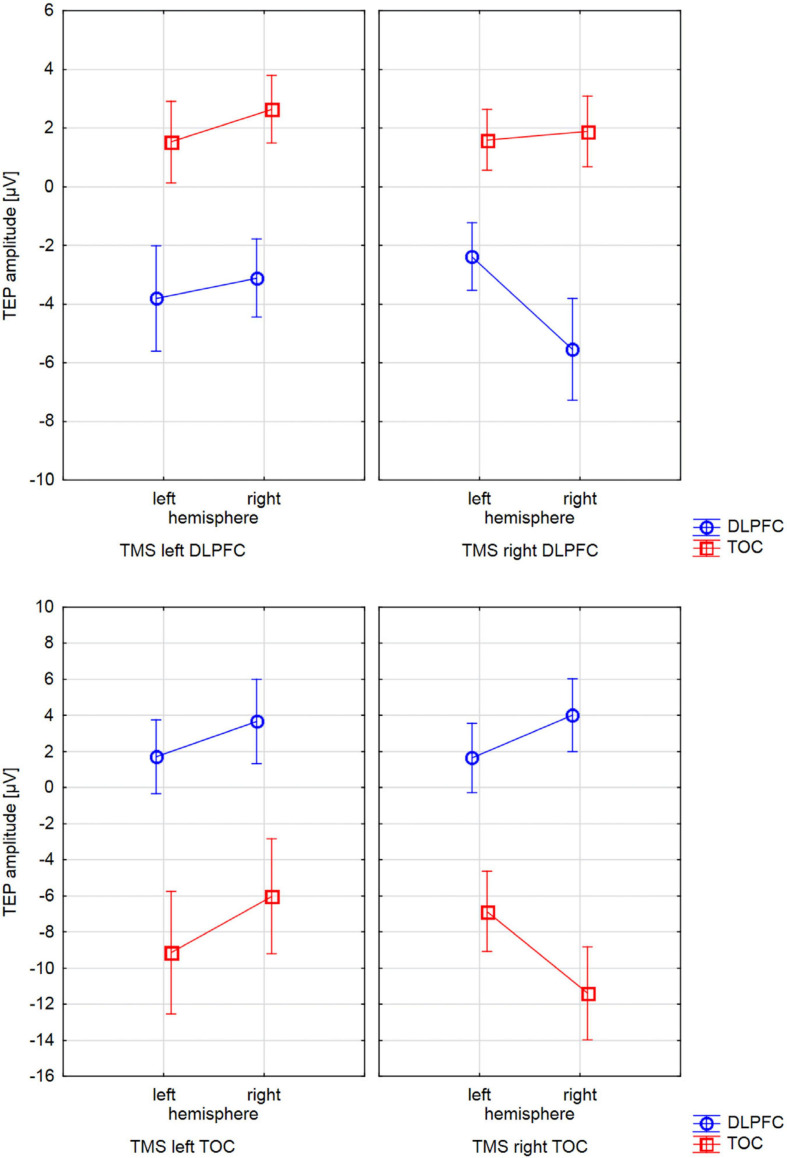
Interactions of TMS SIDE X HEMISPHERE area for all four BRAIN REGIONS. The diagrams present TEP amplitude values at one of the electrodes of interest (TOC left hemisphere: P9, TOC right hemisphere: P10, DLPFC left hemisphere: F5, DLPFC right hemisphere F6). Error bars represent 95% confidence intervals. Each diagram refers to one stimulation condition (i.e., target site), with the upper diagrams presenting left and right DLPFC stimulation and the lower diagrams presenting left and right TOC stimulation. TEP amplitudes at the site of stimulation are lateralized with higher (more negative) amplitudes over the stimulated hemisphere. This effect was statistically significant for the stimulation of the left (*p* = 0.007) and right (*p* = 0.005) temporo-occipital cortex and the right DLPFC (*p* = 0.001). For left DLPFC stimulation, TEPs over the stimulated brain lateralization were not significant. The brain region that was not stimulated did not show systematic lateralization.

For TOC stimulation, in the repeated-measures ANOVA with the dependent variable N180 amplitude and the factors TMS SIDE, HEMISPHERE, and BRAIN REGION, there was a three-way interaction effect TMS SIDE × HEMISPHERE × BRAIN REGION [*F*(1,16) = 18.17; *p* < 0.001; η_*p*_^2^ = 0.53; Figure 2]. There was also a main effect for BRAIN REGION [*F*(1,16) = 48.61; *p* < 0.001; η_*p*_^2^ = 0.75] with higher amplitudes at temporo-occipital electrodes compared to frontal electrodes (Table 1). The results of all effects of the ANOVA are presented in Table 2.

**TABLE 1:** Descriptive values of the N180 and LatTEP N180 component peak latencies and amplitudes in various channels for TMS applied to the temporo-occipital cortex.

Variable	Mean	*SD*
Latency left (ms)	178.8	20.0
F5 left (μV)	1.7	4.0
F6 left (μV)	3.7	4.5
P9 left (μV)	–9.2	6.6
P10 left (μV)	–6.0	6.2
Cz left (μV)	9.4	7.5
Latency right (ms)	183.1	20.4
F5 right (μV)	1.6	3.7
F6 right (μV)	4.0	3.9
P9 right (μV)	–6.9	4.3
P10 right (μV)	–11.4	5.0
Cz right (μV)	10.3	5.4
Latency LatTEP (ms)	171.9	21.6
LatTEP F5/F6 (μV)	0.0	1.6
LatTEP P9/P10 (μV)	–4.3	3.1

*Left and right refer to the respective side of stimulation. SD, standard deviation.*

**TABLE 2:** Results of the repeated-measures ANOVA for TOC stimulation with the dependent variable N180 amplitude.

Effect	**F**	**df**	**p**	η_*p*_^2^
TMS SIDE	0.95	1,16	0.35	0.06
HEMISPHERE	1.26	1,16	0.28	0.07
BRAIN REGION	48.61	1,16	<0.001	0.75
TMS SIDE × HEMISPHERE	9.88	1,16	0.006	0.38
TMS SIDE × BRAIN REGION	0.96	1,16	0.34	0.06
HEMISPHERE × BRAIN REGION	8.66	1,16	0.01	0.35
TMS SIDE × HEMISPHERE × BRAIN REGION	18.17	1,16	0.001	0.53

This three-way interaction was followed up by two-way repeated-measures ANOVAs. As we expected a change of the direction of TEP lateralization at the stimulation site depending on the level of the factor TMS SIDE, these ANOVAs were conducted with the factors HEMISPHERE and BRAIN REGION separately for left-sided TMS and right-sided TMS.

**FIGURE 3:**
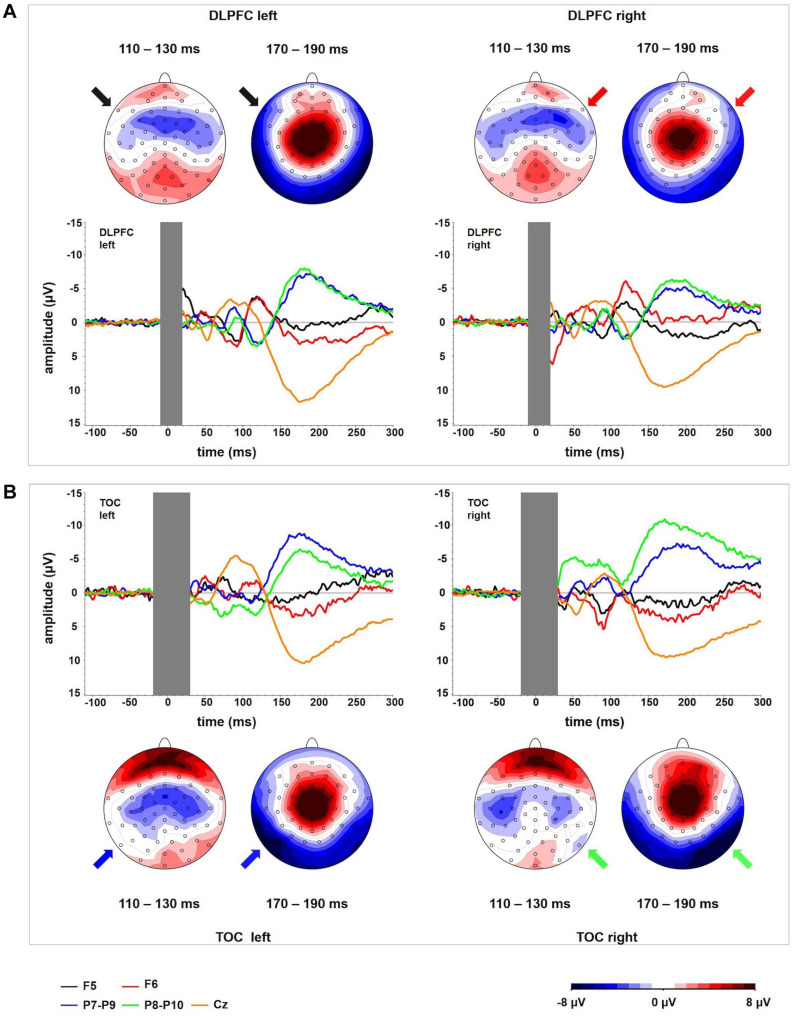
TEP time course for each of the channels corresponding to one of the stimulation locations (F5, F6, P9, P10) and channel Cz. The (top panel, **A**) represents DLPFC stimulation; the (bottom panel, **B**) represents OCC stimulation. The corresponding topographical plots show the time windows in which local stimulation site-specific TEPs peak in each of the stimulation conditions. For DLPFC, there is no activity systematically lateralized toward the stimulated hemisphere in temporo-occipital electrodes in the time window around 180 ms. For TOC, there is no activity systematically lateralized toward the stimulated hemisphere in frontal electrodes around 120 ms. In all conditions, a relatively uniform time course in electrode Cz can be observed.

The two-way ANOVA for stimulation applied to the right side yielded a HEMISPHERE × BRAIN REGION [*F*(1,16) = 30.34; *p* < 0.001; η_*p*_^2^ = 0.66] interaction effect and a main effect for BRAIN REGION [*F*(1,16) = 56.36; *p* < 0.001; η_*p*_^2^ = 0.78]. The main effect is based on higher amplitudes in temporo-occipital electrodes compared to frontal electrodes (Table 1). The two-way interaction was followed up by univariate ANOVAs. In the univariate ANOVA with the factor HEMISPHERE for temporo-occipital electrodes there was a main effect HEMISPHERE [*F*(1,16) = 10.74; *p* = 0.005; η_*p*_^2^ = 0.40], showing that amplitudes were higher in P10 (ipsilateral to TMS) compared to P9 (contralateral to TMS). In the univariate ANOVA with the factor HEMISPHERE for frontal electrodes, there was a main effect for HEMISPHERE [*F*(1,16) = 10.25; *p* = 0.006; η_*p*_^2^ = 0.39], here amplitudes were lower ipsilateral to TMS compared to contralateral. The highest N180 amplitude values for right-sided stimulation were found near the locus of stimulation (ipsilateral temporo-occipital; see Table 1 and Figure 3).

The two-way ANOVA for TMS applied to the left side showed a main effect for HEMISPHERE [*F*(1,16) = 9.47; *p* = 0.007; η_*p*_^2^ = 0.37], which was explained by higher amplitudes over the hemisphere ipsilateral to the side of stimulation (Table 1). Furthermore, there was a main effect for BRAIN REGION [*F*(1,16) = 26.26; *p* < 0.001; η_*p*_^2^ = 0.62], reflecting higher amplitudes at temporo-occipital electrodes compared to frontal electrodes (Table 1). There was no interaction effect for left-sided stimulation. Again, the highest N180 amplitudes were found near the locus of stimulation (ipsilateral temporo-occipital; see Table 1 and Figure 3).

#### Comparison of the N180 Peak Between the Locus of Stimulation and Cz

In the repeated-measures ANOVA with the dependent variable N180 amplitude and the factors TMS SIDE and ELECTRODE LOCALIZATION (factor levels: “ipsilateral temporo-occipital electrode” vs. “Cz”) a main effect for the factor ELECTRODE LOCALIZATION was found [*F*(1,16) = 52.64; *p* < 0.001; η_*p*_^2^ = 0.77]. Amplitudes at Cz were lower than the amplitudes at the ipsilateral temporo-occipital electrodes (site of stimulation; Table 1). There were no other main effects or interaction effects.

#### Comparison of the LatTEP N180 Peaks Across Brain Regions

A univariate repeated-measures ANOVA with the dependent variable LatTEP amplitude and the factor BRAIN REGION (LatTEP F5/F6 vs. LatTEP P9/P10) yielded a main effect [*F*(1,16) = 31.6; *p* < 0.001; η_*p*_^2^ = 0.66]. LatTEPs were higher at parieto-occipital electrodes compared with frontal electrodes (Table 1).

### DLPFC Stimulation

#### TEP Time Course

**FIGURE 4:**
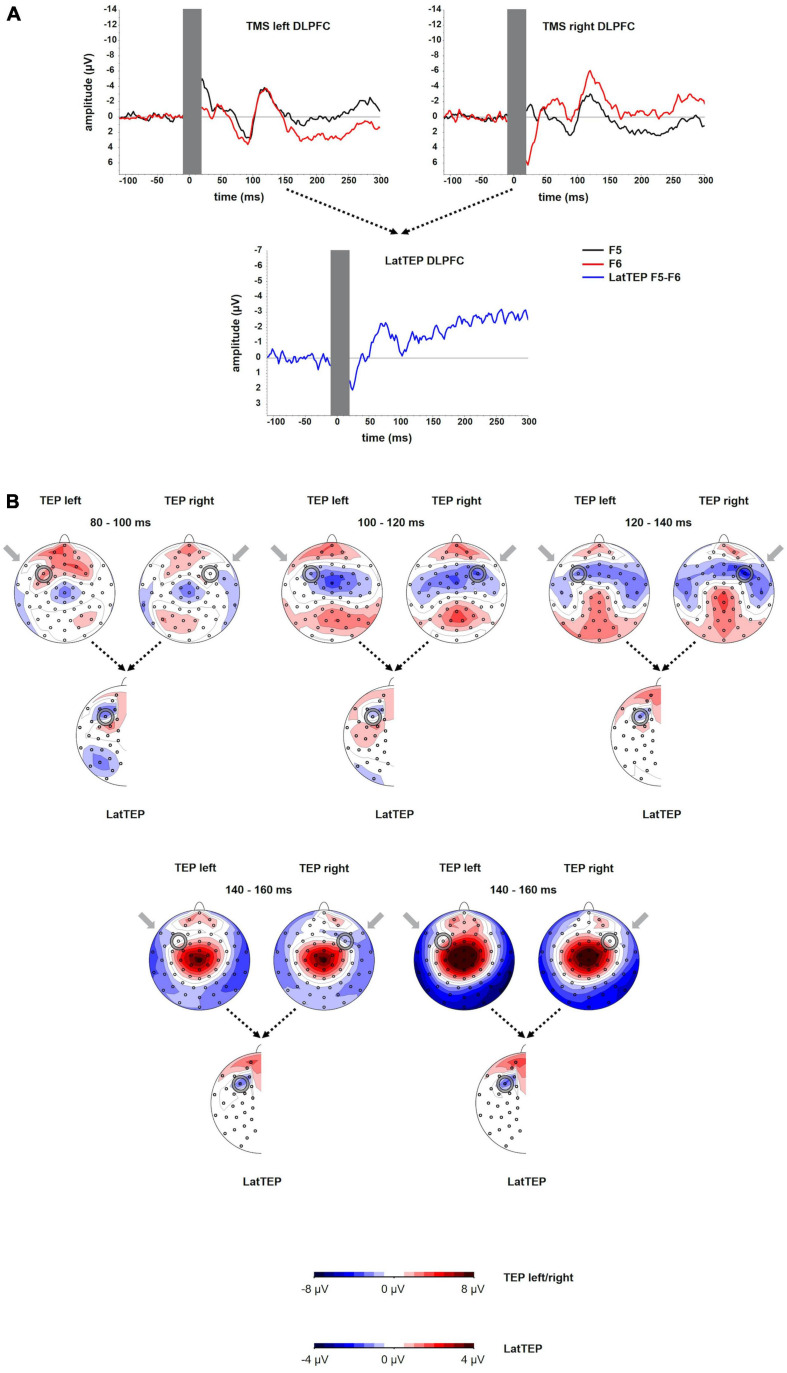
**(A)** TEP time course at electrodes F5 and F6 for TMS to the left (TMS left DLPFC) and the right (TMS right DLPFC) dorsolateral prefrontal cortex. The extent to which TEPs are higher (more negative) ipsilateral than contralateral to the side of stimulation is reflected in LatTEP amplitudes. Lateralization of evoked activity from both stimulation sides is condensed in one measure (LatTEP F5/F6). The LatTEP peaks at approximately 80 ms after the TMS pulse. Note the different scaling of the *y* axis between TEPs and LatTEPs. **(B)** Topographical plots of TEPs in time segments each of 20-ms length for TMS to the left (TMS left DLPFC) and right (TMS right DLPFC) temporo-occipital cortex. LatTEP topographies are derived from TEP maps of both stimulation sides with each channel calculated according to the LatTEP formula. LatTEP maps show a topographical maximum around electrode LatTEP F5/F6 seen most prominently in the time range from 80 to 100 ms. Note that the color-coding scales differ between TEPs and LatTEPs.

The grand averages of the TEPs at electrodes overlying the site of stimulation showed a first negative deflection at approximately 50 ms, a positive deflection peaking at approximately 90 ms and a more prominent negative deflection peaking at approximately 120 ms (N100). The amplitude of the N100 was significantly different from the baseline [at electrode P5 for left TMS: *t*(23) = −4.39; *p* < 0.001; at electrode P10 for right TMS: *t*(16) = −6.60; *p* < 0.001]. The LatTEP curve included a negative deflection with a peak at approximately 80 ms (LatTEP N100; Figure 4A). The LatTEP N100 amplitude at electrode LatTEP F5/F6 was significantly different from the baseline [*t*(23) = −5.72; *p* < 0.001].

### TEP Topography

For right DLPFC stimulation, the topographic distribution showed a distinct negativity around electrode F6 most prominently in the time window 100–140 ms but no apparent lateralized maximum for left DLPFC stimulation. In the time window from approximately 80–120 ms, a central symmetrical negativity (Cz and FCz) was present that resembles the symmetrical negativity found in the corresponding time window of the TOC stimulation. For right DLPFC stimulation, this negativity overlaps and conflates with the lateralized negativity around F6 in the time window from 100 to 120 ms. For left DLPFC stimulation, this negativity extends to both frontal lobes including electrodes F5 and F6 (Figure 4B).

Beginning at a latency of approximately 140 ms, a positivity at the vertex and a posterior bilateral negativity are apparent. The posterior negativity has a slightly asymmetrical topography with a preponderance of the right hemisphere for both left- and right-sided TMS (i.e., the topographic maximum is not systematically located in the stimulated hemisphere). Its topographic distribution corresponds to the pattern seen in TOC stimulation except for the additional systematically lateralized activity around P9 and P10 observed in TOC stimulation.

In LatTEP maps, symmetrical evoked activity and also asymmetrical activity that is not systematically lateralized with respect to the side of stimulation are canceled out. Consequently, a negativity with a maximum at electrode LatTEP F5/F6 is visible most prominently in the time window from 80 to 100 ms (Figure 4B).

#### Lateralized Site-Specific Activity at the Stimulation Site for DLPFC TMS

For DLPFC stimulation, the repeated-measures ANOVA with the dependent variable N100 amplitude and the factors TMS SIDE, HEMISPHERE, and BRAIN REGION showed a strong trend toward a three-way interaction effect TMS SIDE × HEMISPHERE × BRAIN REGION [*F*(1,23) = 4.05; *p* = 0.056; η_*p*_^2^ = 0.15]. Furthermore, there was a main effect for BRAIN REGION [*F*(1,23) = 59.37; *p* < 0.001; η_*p*_^2^ = 0.72] due to higher amplitudes in frontal compared to temporo-occipital electrodes (Table 3). The results of all effects of the ANOVA are presented in Table 4.

**TABLE 3:** Descriptive values of the N100 and LatTEP N100 component peak latencies and amplitudes in various channels for TMS applied to the dorsolateral prefrontal cortex.

Variable	Mean	**SD**
Latency left (ms)	115.9	15.3
F5 left (μV)	–3.8	4.2
F6 left (μV)	–3.1	3.2
P9 left (μV)	1.5	3.3
P10 left (μV)	2.6	2.7
Cz left (μV)	0.4	3.1
Latency right (ms)	113.7	16.1
F5 right (μV)	–2.4	2.7
F6 right (μV)	–5.5	4.1
P9 right (μV)	1.6	2.5
P10 right (μV)	1.9	2.9
Cz right (μV)	0.7	3.8
Latency LatTEP (ms)	83.8	20.0
LatTEP F5/F6 (μV)	–2.6	3.3
LatTEP P9/10 (μV)	–0.8	1.6

*Left and right refer to the side of stimulation. SD, standard deviation.*

**TABLE 4:** Results of the repeated-measures ANOVA for DLPFC stimulation with the dependent variable N100 amplitude.

Effect	**F**	**df**	**p**	η_*p*_^2^
TMS SIDE	1.55	1,23	0.23	0.06
HEMISPHERE	0.02	1,23	0.50	0.02
BRAIN REGION	59.47	1,23	< 0.001	0.72
TMS SIDE × HEMISPHERE	9.76	1,23	0.005	0.30
TMS SIDE × BRAIN REGION	0.03	1,23	0.87	0.001
HEMISPHERE × BRAIN REGION	5.66	1,23	0.026	0.20
TMS SIDE × HEMISPHERE × BRAIN REGION	4.05	1,23	0.056	0.15

As the trend toward a three-way interaction is consistent with our *a priori* hypothesis, we used two-way repeated-measures ANOVAs to follow up this interaction. Again, as a change of the direction of TEP lateralization at the stimulation site depending on the level of the factor TMS SIDE was expected, these ANOVAs were conducted with the factors HEMISPHERE and ELECTRODE separately for left-sided TMS and right-sided TMS.

The two-way ANOVA for TMS applied to the left DLPFC yielded a main effect for BRAIN REGION [*F*(1,23) = 39.09; *p* < 0.001; η_*p*_^2^ = 0.63]; no other main effects or interaction effects were found. TEP amplitudes were higher at frontal electrodes than at temporo-occipital electrodes. The descriptively highest N100 amplitude was found over the DLPFC ipsilateral to TMS (Table 3 and Figure 3); however, lateralization was not significant in this condition.

The two-way ANOVA for TMS applied to the right DLPFC showed a main effect for HEMISPHERE [*F*(1,23) = 13.86; *p* = 0.001; η_*p*_^2^ = 0.38], a main effect for BRAIN REGION [*F*(1,23) = 42.57; *p* < 0.001; η_*p*_^2^ = 0.65], and a HEMISPHERE × BRAIN REGION interaction [*F*(1,23) = 9.48; *p* = 0.005; η_*p*_^2^ = 0.29]. In order to further elucidate this interaction effect, we performed univariate repeated-measures ANOVAs with the factor HEMISPHERE separately for frontal and temporo-occipital electrodes.

In the univariate ANOVA with the factor HEMISPHERE for frontal electrodes, there was a main effect [*F*(1,23) = 15.96; *p* = 0.001; η_*p*_^2^ = 0.41] explained by higher amplitudes over the stimulated hemisphere compared to the contralateral hemisphere (Table 3). In the univariate ANOVA with the factor HEMISPHERE for temporo-occipital electrodes, no main effect was found [*F*(1,23) = 2.84; *p* = 0.60; η_*p*_^2^ = 0.01]. The highest N100 amplitude was found at the site of stimulation (ipsilateral frontal electrode; Table 3 and Figure 3).

#### Comparison of the N100 Between the Locus of Stimulation and Cz

In a repeated-measures ANOVA with the factors TMS SIDE and ELECTRODE LOCALIZATION (factor levels: “electrode at the site of stimulation” and “electrode Cz”), there was a main effect for the factor ELECTRODE LOCALIZATION [*F*(1,23) = 14.60; *p* = 0.001; η_*p*_^2^ = 0.39]. N100 amplitudes were higher at the site of stimulation compared to at Cz (Table 3). No other main effects or interaction effects were found.

#### Comparison of the LatTEP N100 Peak Across Brain Regions

In a univariate repeated-measures ANOVA with the dependent variable LatTEP N100 amplitude, we found a significant main effect of BRAIN REGION [levels: LatTEP F5/F6 and LatTEP P9/P10; *F*(1,23) = 6.70; *p* = 0.016; η_*p*_^2^ = 0.23], with higher LatTEP N100 amplitudes at frontal electrodes.

## Discussion

The major findings of the study were that TEPs evoked by TMS to the TOC and the DLPFC contained systematically lateralized negative long-latency components over the stimulated brain region that most likely reflect transcranial TMS effects on the targeted cortex area. It was possible to isolate lateralized activity at the stimulation site in LatTEPs by stimulating homologous sites in both hemispheres and subtracting invariable evoked activity, an approach that can improve TEP methodology in future studies aiming to assess local cortical functions.

### LatTEP Components at the Stimulation Site

We specifically searched for evoked components with long-latency ranges and a lateralized ipsilateral topography because components with lateralized topography confined to the site of stimulation are most likely not a correlate of unspecific processes (Conde et al., 2019). Our hypothesis predicted that TEP amplitudes in the stimulated brain region would be systematically higher ipsilateral to TMS than contralateral to TMS. In all stimulation conditions, the highest amplitudes were systematically found over the stimulation site. TEP peak amplitudes in the stimulated brain region were lateralized with higher amplitudes over the stimulated hemisphere in three of four conditions. For TMS over the left DLPFC, the N100 amplitude was also descriptively higher in ipsilateral compared to that in contralateral electrodes, but the difference did not surpass the threshold of statistical significance possibly due to low sample size and measurement error. In agreement with our hypothesis, no systematic lateralization toward the side of stimulation was found in electrodes outside the stimulated brain region (e.g., frontal electrodes for TOC TMS).

### Isolating Lateralized Activity in LatTEPs

To eliminate evoked activity, which was not systematically lateralized to the side of TMS, we adopted the methodology of the LRP (Coles, 1989), which, to our knowledge, has not been applied to TEPs before. Lateralized negativity at the site of stimulation that may be masked by symmetrical processes in conventional maps can be unmasked in LatTEP topoplots (e.g., Figure 1B in time window 120–140 ms). In TOC stimulation, a prominent lateralized negativity was found with a topographic maximum around electrode LatTEP P9/P10 (Figure 1B); in DLPFC stimulation, there was a negative maximum located over the targeted brain region around electrode LatTEP F5/F6 (Figure 4B). The statistical comparison of LatTEP peaks across the two brain regions corroborated the results found for conventional TEPs that higher LatTEP amplitudes can be found in the stimulated compared to the non-stimulated brain region for both TOC and DLPFC stimulation. It is noteworthy that LatTEP negativity can result from ipsilateral negative voltages and contralateral positive voltages. Therefore, the interpretation of LatTEPs needs to take into account the original time course and topography of TEPs of both sides. As there was no prominent positivity contralateral as a potential cause of the negative LatTEP maxima, they are caused by a negativity in ipsilateral electrodes surrounding the target site.

### Do Lateralized Site-Specific Components Represent Transcranially Evoked Activity?

Although lateralized components specific to the stimulation site likely reflect direct transcranial effects of TMS (Conde et al., 2019), potential alternative explanations include decay artifacts, which are commonly observed close to the site of stimulation. These artifacts result from an initial quick polarization of the electrode contact by the TMS pulse and a subsequent continuous discharge. The time course of decay artifacts is highly consistent across trials and individuals with a peak within the first 10–50 ms followed by an exponential decay of the voltage (Rogasch et al., 2014; Ilmoniemi et al., 2015). Thus, the time course of the lateralized components observed in our study with a slow deflection beginning at approximately 100 ms is not compatible with a decay artifact.

A second alternative explanation may be artifacts related to muscle twitches, which can be mostly observed when stimulating in the vicinity of cranial muscles. These artifacts present with very high amplitudes (10–1,000 μV) have a biphasic course with a positive and a negative peak occurring within the first 20 ms and last up to a maximum of 60 ms. The topography is reminiscent of a tangential dipole with adjacent positive and negative poles (Mutanen et al., 2013; Rogasch et al., 2014). In this case, not only the time course but also the amplitude and topography are incompatible with muscle twitches. Thus, we consider transcranially evoked activity in the targeted cortex area to be the most likely origin of the lateralized late components.

While to our knowledge previous studies have not assessed the extent of lateralization of TEPs in an approach similar to ours, our results are nevertheless compatible with the results of some previous TMS-EEG studies. TEP topographies with maxima located over the stimulated hemisphere in the vicinity of the stimulation site can often be found in studies targeting M1 (Paus et al., 2001; Bonato et al., 2006; Bruckmann et al., 2012; Yamanaka et al., 2013; Jarczok et al., 2016). However, in TMS-EEG investigations targeting other brain areas, such topographies were found at short latencies but not at long latencies (Rogasch et al., 2014; Herring et al., 2015; Noda et al., 2016; Du et al., 2017). Because of smaller amplitudes of transcranially evoked components in DLPFC stimulation, lateralized components may be overshadowed by central unspecific activity more easily than in M1 stimulation. Calculation of LatTEPs may be useful to uncover LatTEP components masked by more prominent non-lateralized components.

### Non-specific Evoked Components Overlap With Transcranially Evoked Components

In all four stimulation conditions, invariable components overlapping with site-specific components were observed. Topographies across all stimulation conditions display a symmetrical negativity with a maximum at the vertex (time range from 80 to 120 ms; Figures 1B, 4B), and a symmetrical positivity with a maximum at the vertex co-occurring with a bilateral temporo-occipital negativity is (140–180 ms; Figures 1B, 4B). A uniform time course in electrode Cz was found with a negative peak at approximately 100 ms and a positive peak at approximately 180 ms (Figure 3) for all conditions. Because of the shorter latency and the significantly lower peak amplitude compared to the lateralized site-specific negative peaks, lateralized components cannot be explained by volume conduction from the process observed at Cz.

As we intended to identify local activity specific to the stimulated cortical site in the presence of sensory-evoked potentials, no masking procedure was applied. The spatiotemporal pattern of the non-specific component is compatible with an auditory evoked potential (AEP), which is characterized by a N100-P180 complex with a frontocentral, mostly symmetrical topography (Hine and Debener, 2007; Mahajan and McArthur, 2012; Lightfoot, 2016). Additionally, somatosensory-evoked potentials (SSEPs) present with deflections with similar latencies (N140, P190) and contralateral or bilateral maxima over somatosensory areas (Goff et al., 1977; Allison et al., 1992; Genna et al., 2016) that likely contribute to the overall topography of TEPs. However, given their known topography, AEPs and SSEPs cannot be the underlying causes of ipsilateral LatTEP components. AEPs are mostly symmetrical in binaural stimulation or can present with lateralized late negative AEP components (N1) with higher amplitudes over the contralateral hemisphere (McCallum and Curry, 1980; Hine and Debener, 2007). Late negative SSEP components also present with higher contralateral amplitudes (Hashimoto, 1988; Genna et al., 2016). Additionally, sensory-evoked potentials are generated in cortical areas specific to the respective sensory modality. A shift of the topographic maximum to the stimulated brain region when the target site changes are not compatible with AEPs or SSEPs.

Our results are in agreement with the findings of a comparison of TMS with a sensory stimulation, in which the most prominent difference between the two stimulation conditions at long latencies was observed in electrodes close to the stimulation site. A principal component analysis revealed a component consistent with lateralized activity over the stimulated cortex area that explained approximately 59% of the variance only in the real TMS condition. In both conditions, there were components compatible with a non-lateralized central N100-P180 complex (Biabani et al., 2019). Together with our findings, this is consistent with the notion that transcranially evoked components can be found over the site of stimulation, whereas potentials at other sites are substantially confounded by sensory input. Understanding the composition of TEPs is particularly relevant, as it may not be possible to eliminate sensory confounders completely with current procedures (ter Braack et al., 2015; Biabani et al., 2019; Siebner et al., 2019).

### Latencies in TOC and DLPFC Stimulation

Latencies of the late negative peaks at the site of stimulation varied substantially across brain regions but were consistent across hemispheres within one brain region. A systematic evaluation of DLPFC latencies at electrodes close to the locus of stimulation reported mean latencies of approximately 110 to 115 ms (Lioumis et al., 2009) well compatible with our results (approximately 115 ms). Posterior cortex areas are less well characterized, and we are not aware of studies that systematically investigated the variance of latencies and amplitudes of TEPs in the temporal or occipital cortex. However, the data of several previous studies are compatible with markedly longer latencies in posterior cortex areas (Rosanova et al., 2009; Herring et al., 2015; Samaha et al., 2017; Belardinelli et al., 2019), although some reported conflicting results (Kerwin et al., 2017). Our findings suggest that the second prominent negative TEP peak in TOC TMS has a latency of approximately 170–180 ms.

A direct statistical comparison of DLPFC and TOC stimulation latencies in our study is not possible because of methodological differences. However, the difference between groups of approximately 4 standard deviations of the mean DLPFC latency most likely reflects that TEPs differ substantially across different stimulated cortical areas (Kähkönen et al., 2005; Lioumis et al., 2009; Casarotto et al., 2010).

### Neurobiological Processes Associated With the Generation of TEPs

Transcranial magnetic stimulation causes synchronized depolarization in pyramidal cells and interneurons (Di Lazzaro and Ziemann, 2013) and consequentially fluctuations of excitatory postsynaptic potentials in the targeted cortex. Therefore, local TMS-evoked activity generated by the targeted population of neurons can be expected to be found at the stimulated cortex site. However, after the initial activation of local neurons, secondary activation of other (potentially remote) cortical and subcortical structures occurs that is not fully understood. Our results add evidence that not only short latency but also long-latency transcranially evoked components generated by the stimulated cortical region can be found in the compound TEP.

Based mostly on experiments targeting M1 the N100 component has been linked to inhibitory activity (Nikulin et al., 2003; Bender et al., 2005; Bruckmann et al., 2012). Pharmacological interventions point to an involvement of GABA-B-ergic neutrotransmission (Premoli et al., 2014). In agreement with our findings, pharmacological effects of GABA-B agonist baclofen were found close to the stimulation site but not at remote electrodes. Despite the differences in latencies between TOC and DLPFC, late components may reflect GABA-ergic neurotransmission as the latency of GABA-B–associated inhibitory postsynaptic potentials varies, substantially depending on properties of the local neurons (Thomson and Destexhe, 1999). However, experiments such as pharmacological challenges (Premoli et al., 2014) would be necessary to further elucidate the underlying neurobiology of TEPs outside M1. Our results suggest that researchers should also specifically consider TEP components located over the targeted brain area and lateralized toward the stimulated hemisphere when further investigating TEPs.

### Limitations

Temporo-occipital cortex and dorsolateral prefrontal cortex stimulations were applied to separate groups of subjects. Thus, a direct comparison of absolute values or within-subject comparisons of variables across the two stimulation sites is not possible. However, the different samples and methodological differences cannot account for the effects of hemispheric lateralization and the stimulation site-specific topographies of evoked activity found across all conditions. We argue that the finding of evoked activity at the site of stimulation despite these differences supports the generalizability and robustness of the results.

## Conclusion

The results of the present study show that TEPs contain long-latency negative components that are lateralized toward the stimulated hemisphere and have their topographic maxima at the respective stimulation sites. Removing not systematically lateralized evoked activity by calculating LatTEPs reduced overshadowing by unspecific components and revealed negative maxima located around the target sites. The systematic lateralization and the localization at the stimulation site suggest that these components are correlates of cortical activity evoked directly by local effects of the magnetic field. Clinical and research applications of TEPs can benefit from specifically focusing on LatTEP components at the stimulation site.

## Data Availability Statement

The datasets presented in this article are not readily available because no permission to transfer the data of individual subjects to third parties has been granted by the local ethics committees. Requests to access the datasets should be directed to TJ, tomasz.jarczok@uk-koeln.de.

## Ethics Statement

The studies involving human participants were reviewed and approved by Ethics Committee of the Faculty of Medicine, University Hospital Cologne, Cologne, Germany and Ethics Committee of the Technical University Dresden, Dresden, Germany. The patients/participants provided their written informed consent to participate in this study.

## Author Contributions

TJ contributed to data analysis, statistical analysis, interpretation and visualization, supervision of data acquisition, and wrote the original manuscript draft. FR contributed to data acquisition and data analysis. LP and LB contributed to data acquisition and edited the manuscript. VR contributed resources and edited the manuscript. CK contributed to writing the manuscript. SB contributed to the experimental design, resources, supervision, and edited the manuscript. All the authors contributed to the article and approved the submitted version.

## Conflict of Interest

VR has received payment for consulting and writing activities from Lilly, Novartis, and Shire Pharmaceuticals, lecture honoraria from Lilly, Novartis, Shire Pharmaceuticals/Takeda, and Medice Pharma, support for research from Shire Pharmaceuticals/Takeda and Novartis, and also carried out clinical trials in cooperation with the Novartis, Shire Pharmaceuticals/Takeda, Servier, and Otsuka companies. SB received support for symposia by Shire/Takeda, Actelion, and Medice Pharma as well as honoraria from Roche and Medic ePharma. The remaining authors declare that the research was conducted in the absence of any commercial or financial relationships that could be construed as a potential conflict of interest.

## Footnotes

1 https://www.clinicalresearcher.org/software.htm
