# Supplementary figures and images for: Single-Pulse TMS to the Temporo-Occipital and Dorsolateral Prefrontal Cortex Evokes Lateralized Long Latency EEG Responses at the Stimulation Site
Source: Front Neurosci. 2021 Mar 12;15:616667. doi: 10.3389/fnins.2021.616667 (PMC8006291; doi:10.3389/fnins.2021.616667)

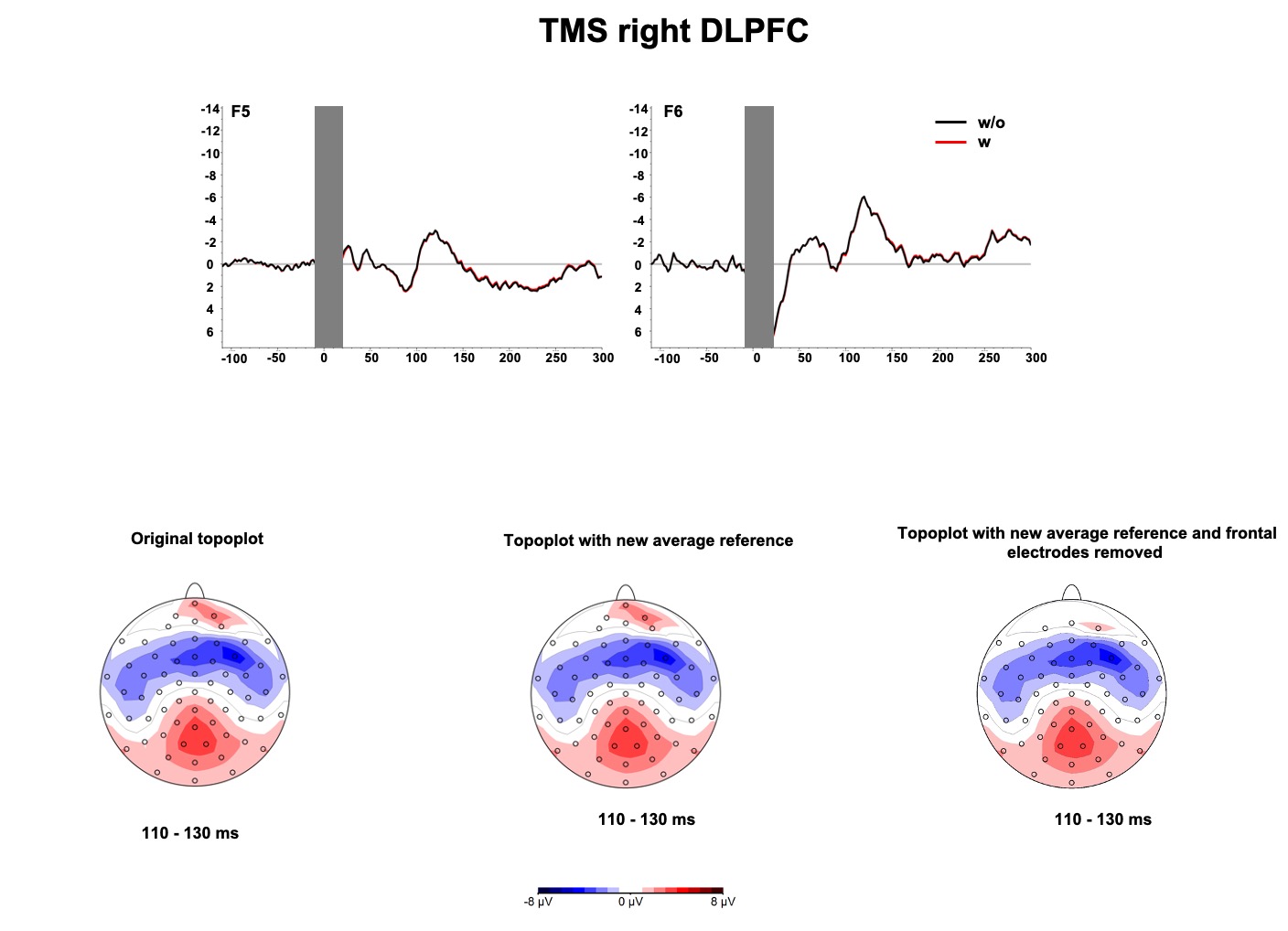

Supplement: Supplementary file 1 [file Image_1.JPEG]
