# Supplementary material for: Single-Pulse TMS to the Temporo-Occipital and Dorsolateral Prefrontal Cortex Evokes Lateralized Long Latency EEG Responses at the Stimulation Site
Source: Front Neurosci. 2021 Mar 12;15:616667. doi: 10.3389/fnins.2021.616667 (PMC8006291; doi:10.3389/fnins.2021.616667)
Supplement: Supplementary file 2 [file Presentation_1.pdf]

Supplementary material

1. Procedure for determining the stimulation site for temporo-occipital cortex TMS

The exact site of stimulation for temporo-occipital cortex stimulation was determined by localizing the visual N700 event-related potential (ERP) component (Bender et al., 2008). To this end subjects performed a visual working memory task (change detection task) before the transcranial magnetic stimulation (TMS) procedure. In this task an initial visual stimulus (S1) consisting of colored squares distributed equally over both visual hemifields (5 in each hemifield) had to be compared to a second stimulus (S2). S2 was presented to either the right or the left visual hemifield. In 50% of trials S2 was identical to the same hemifield presented in S1. Subjects were instructed to indicate whether S2 differed from the corresponding hemifield in S1 or not by pressing one of two buttons. A version of task with minor variations is described in detail by Hecht et al. (2016). The differences to that task were a shorter interval between S1 and S2 (900 ms), less trials (32 stimulus pairs) and a shorter inter-stimulus interval (varying between 6 and 10 ms) in the task used here. ERPs were analysed immediately after with Brain Vision Analyzer 2.1 software (Brain Products GmbH, München) in order to determine the electrode with the highest N700 peak amplitude in each individual participant, which was then chosen as the stimulation site for this person. The locus of stimulation was determined separately for each hemisphere. Therefore, S2 was averaged separately for left and the right visual hemifield presentation. The visual N700 component has its modality specific maximum amplitude contralateral to the side of the presentation of S2 over the visual association cortex (Bender et al., 2010). If no N700 component was detectable in a participant, TMS was performed at electrodes P9 for the left hemisphere and P10 for the right hemisphere.

2. Effects of the exact coil position on TEP topography

TMS was administered at electrodes P7 (n=4), P9 (n=7), between P9 and P11 (n=2) or at P11 (n=4) for TMS to the left temporo-occipital cortex (TOC) and at P8 (n=3), P10 (n=3) between P10 and P12 (n=5) or at P12 (n=6). TEP grand averages calculated separately for each exact coil location in the temporo-occipital cortex (TOC) stimulation condition revealed only a small non-systematic effect on the topography. The lateralization effect found for the overall group with higher N180 amplitudes ipsilateral

to the stimulated hemisphere was present in each of the sub-groups (Supplementary Figure 1). LatTEPs on a single subject level show a robust negative maximum at temporo-occipital electrodes in individual averages (examples in Supplementary Figure 2).

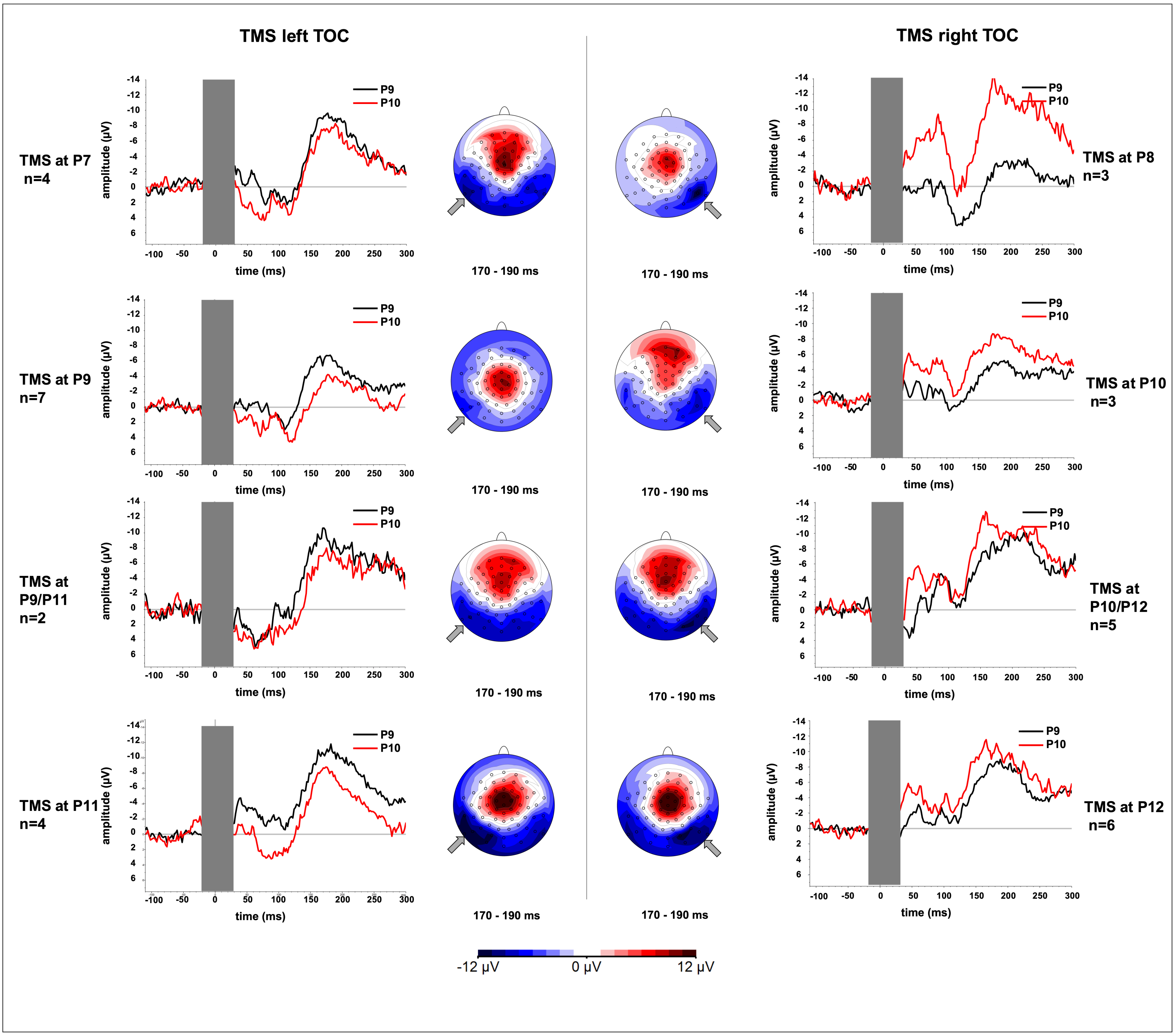

Supplementary Figure 1: TEP grand averages for each sub-group that was stimulated at a specific location in the temporo-occipital cortex. In each of the sub-groups TEP time courses at electrodes P9 and P10 show higher N180 amplitudes in the electrode ipsilateral to the stimulated hemisphere (i.e., P9 higher than P10 for TMS to the left temporo-occipital cortex, P10 higher than P10 for TMS to the right temporo-occipital cortex). A negative ipsilateral maximum can be observed in the corresponding topoplots in temporo-occipital electrodes in the time window 170 to 190 ms.

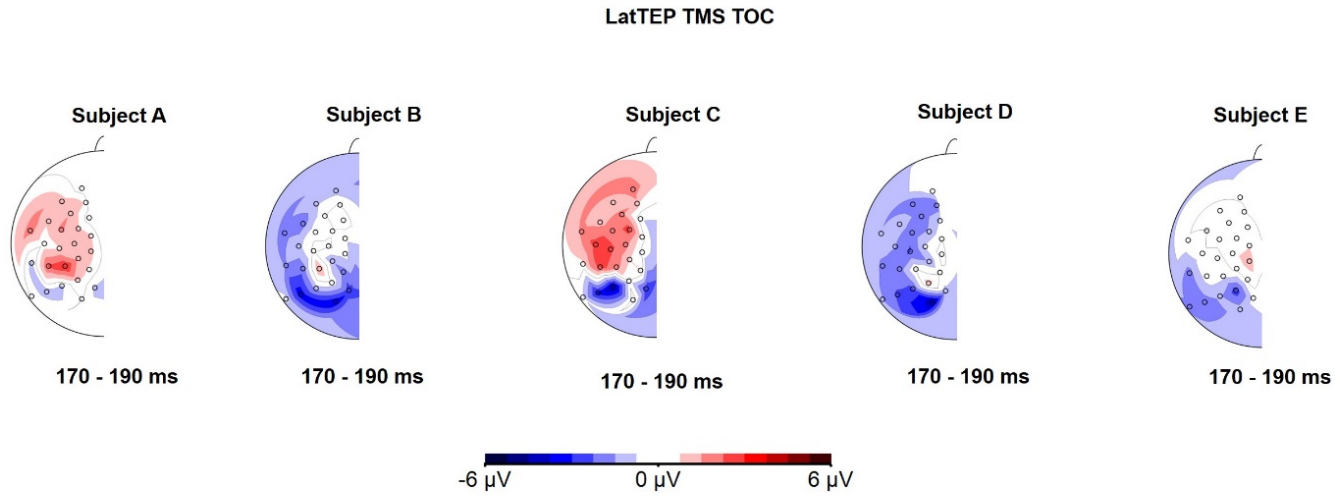

Supplementary Figure 2: Examples of individual LatTEP averages for five subjects. The negative maximum in electrodes around the stimulation site in the temporo-occipital cortex can be observed on a single subject level.

3. Assessment of Reliability

Supplementary Table 1: Intraclass correlation of the peak amplitudes of TEP averages of odd and even trials.

Electrode (Stimulation Site)	ICC	CI 95%	
F5 (DLPFC left)	0.89	0.76	0.95
F6 (DLPFC right)	0.95	0.89	0.98
P9 (TOC left)	0.98	0.93	0.99
P10 (TOC right)	0.78	0.42	0.92

ICC intraclass correlation coefficient

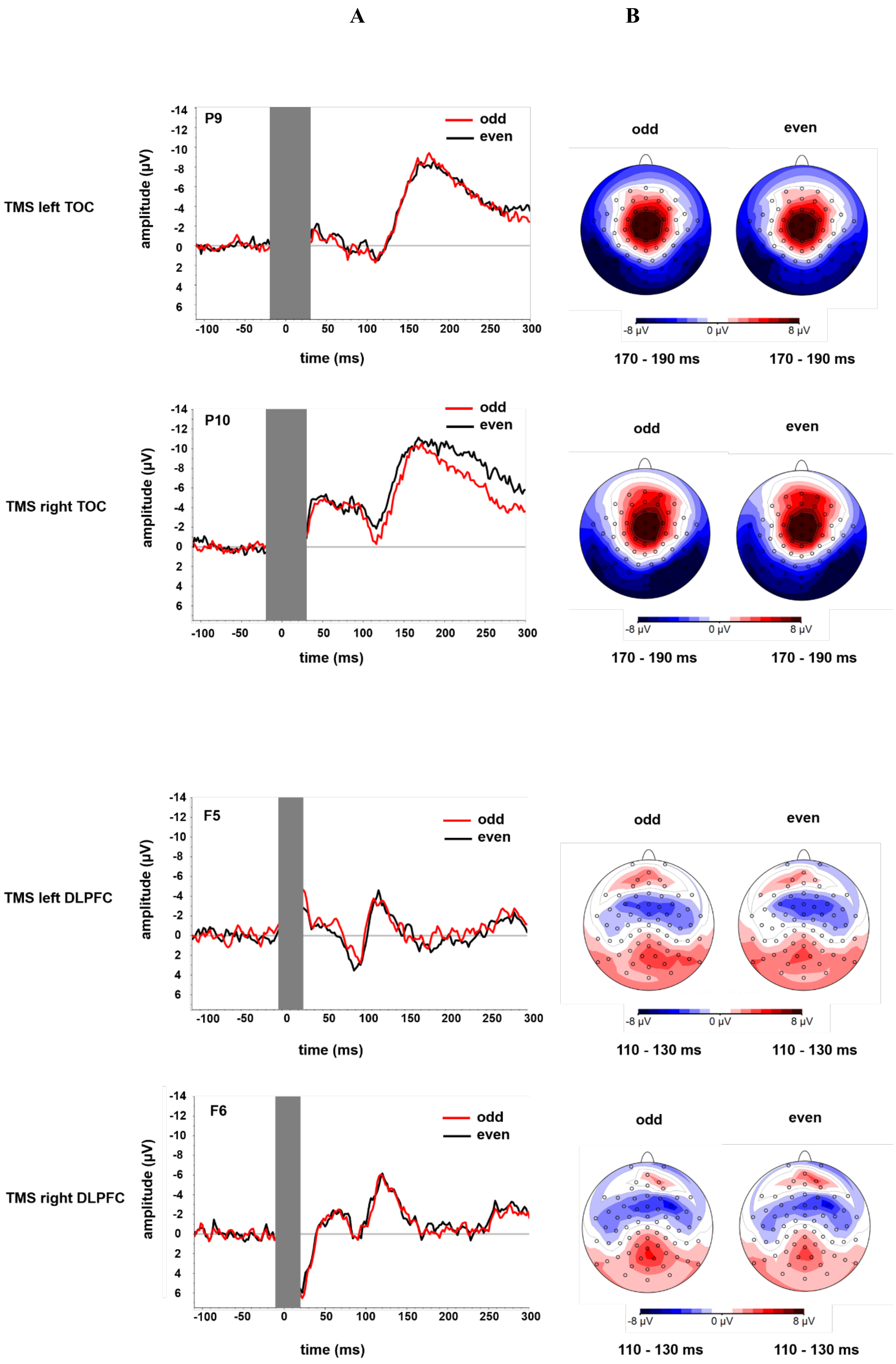
A
B
TMS left TOC
P9
odd
even
amplitude (µV)
time (ms)
170 - 190 ms
-8 µV
0 µV
8 µV
TMS right TOC
P10
TMS left DLPFC
F5
110 - 130 ms
TMS right DLPFC
F6

Supplementary Figure 3: Time course of TEPs at the site of stimulation (A) and topoplots (B) for split-half averages in each stimulation condition. Odd and even trials were averaged separately to assess the reliability of the measurements. Topoplots present the time window corresponding to the peak latency of the TEP in the respective condition. A high reliability was found across the time course of the TEP and in particular at the latency oft long latency negative peak.

References:

Bender, S., Behringer, S., Freitag, C.M., Resch, F., Weisbrod, M., 2010. Transmodal comparison of auditory, motor, and visual post-processing with and without intentional short-term memory maintenance. Clin. Neurophysiol. 121, 2044–2064.

Bender, S., Oelkers-Ax, R., Hellwig, S., Resch, F., Weisbrod, M., 2008. The topography of the scalp-recorded visual N700. Clin. Neurophysiol. 119, 587–604.

Hecht, M., Thiemann, U., Freitag, C.M., Bender, S., 2016. Time-resolved neuroimaging of visual short term memory consolidation by post-perceptual attention shifts. NeuroImage 125, 964–977.
